# Forward with Dementia: process evaluation of an Australian campaign to improve post-diagnostic support

**DOI:** 10.1186/s12913-023-10347-4

**Published:** 2023-12-07

**Authors:** Lee-Fay Low, Meredith Gresham, Lyn Phillipson, Yun-Hee Jeon, Danika Hall, Amy Tan, Nora Wong, Henry Brodaty

**Affiliations:** 1 School of Health Sciences, Faculty of Medicine and Health, University of Sydney, Sydney, Australia; 2 Centre for Healthy Brain Ageing, Discipline of Psychiatry and Mental Health, School of Clinical Medicine, University of New South Wales, Sydney, Australia; 3 School of Health and Society, Faculty of the Arts, Social Sciences and Humanities, University of Wollongong, Wollongong, Australia; 4 Sydney Nursing School, Faculty of Medicine and Health, University of Sydney, Sydney, Australia

**Keywords:** Dementia, Alzheimer’s disease, Post-diagnostic support, Process evaluation, Health care professionals

## Abstract

**Background:**

Forward with Dementia is a co-designed campaign to improve communication of dementia diagnosis and post-diagnostic support.

**Methods:**

Webinars, a website, social and traditional media, and promotions through project partners were used to disseminate campaign messages to health and social care professionals (primary audience) and people with dementia and carers (secondary audience). The campaign ran between October 2021 and June 2022, with 3-months follow-up. The RE-AIM framework was used for process evaluation. Measurements included surveys and interviews, a log of activities (e.g. webinars, social media posts) and engagements (e.g. attendees, reactions to posts), and Google Analytics.

**Results:**

There were 29,053 interactions with campaign activities. More than three-quarters of professionals (*n* = 63/81) thought webinars were very or extremely helpful. Professionals and people with dementia and carers reported that the website provided appropriate content, an approachable tone, and was easy to use. Following campaign engagement, professionals planned to (*n* = 77/80) or had modified (*n* = 29/44) how they communicated the diagnosis and/or provided post-diagnostic information and referrals. Qualitative data suggested that the campaign may have led to benefits for some people with dementia and carers.

**Conclusions:**

Forward with Dementia was successful in terms of reach, appropriateness, adoption and maintenance for professionals, however flow-through impacts on people with dementia are not clear. Targeted campaigns can potentially change health professionals’ communication and support around chronic diseases such as dementia.

## Background

Support in the period immediately following dementia diagnosis is an area of unmet need for people with dementia and carers in many countries [1]. Ideal support after diagnosis is timely and integrated, and includes management of dementia, psychological and emotional wellbeing and practical support [2]. Internationally few examples exist of comprehensive post-diagnostic services delivered consistently across a region [3]. In Australia there are major gaps in post-diagnostic supports [4]. Memory clinics provide limited follow-up and minimal functional or psychological interventions [5].

When people with dementia and carers do not receive adequate post-diagnostic support, they may not know how to best manage their dementia, may not be receiving optimal treatment, and may feel lost or hopeless [6]. Reasons for not getting sufficient post-diagnostic support include reluctance to use services and supports [7], difficulty accessing services [8, 9], and insufficient provision of services [5, 10–12]. Therapeutic nihilism and stigma amongst health professionals and the public are also barriers to provision and accessing supports [13, 14].

The World Health Organisation policy brief on a human rights approach to dementia emphasised the rights of people living with dementia to participate in health decision-making and the importance of empowerment and supporting their autonomy [15]. More than 85% of people would prefer to know their diagnosis if they have dementia, believing that this knowledge would help them stay independent and plan their life [16]. Reasons people do not want to know are fear of or being upset by the diagnosis, and lack of benefits from having the diagnosis [16]. Hence it is important that diagnosticians sensitively tell people with dementia their diagnosis and offer hope and follow-up, in order to help people with dementia and their carers adjust to and manage the diagnosis [17].

Previous dementia mass media campaigns with published evaluations conducted in Australia, the Netherlands and Belgium have focused on dementia risk reduction. These campaigns successfully increased some aspects of knowledge around dementia risk reduction, though they did not assess actual behavioural change [18–20]. All three campaigns used mass and social media along with a website. In addition, the Dutch campaign included community participation and activities such as workshops and lectures in three geographic regions – community participation resulted in better recognition of campaign material and the website [20]. In contrast, the dementia friendly communities are regional grassroots, community-based campaigns to support people with dementia and carers within that region [21, 22]. The campaigns typically involve people living with dementia and carers, inclusive environmental design and public education to reduce stigma and raise awareness [21, 22].

The overarching aim of this project was to improve post-diagnostic support in the 12 months after dementia diagnosis for people living with dementia and carers in Australia, United Kingdom (UK), Canada, the Netherlands and Poland. In Australia, the campaign focused on changing health and social care professional behaviour around communicating the diagnosis and provision of post-diagnostic support information on adjustment and management of dementia and available services for people with dementia and family carers. This paper reports on the process evaluation of the Australian campaign.

## Methods

### The Forward with Dementia campaign

#### Brand and campaign development

The campaign was informed by interviews and surveys with people diagnosed with dementia and carers from Australia, UK, Canada, the Netherlands and Poland, and a review of past and current dementia public health campaigns. The brand and website were co-designed with assistance of a marketing company and input and feedback from people living with dementia, carers, health and social care professionals and key stakeholders (e.g. peak body representatives, policy makers) in the five countries. Australian co-designers and stakeholders had further input into the design of the Australian campaign including diagnosticians and providers of post-diagnostic support. We emphasised the viewpoints and voices of people with dementia and carers throughout. Website user testing was undertaken [23].

We designed an exclusively online campaign given COVID-19 restrictions. The campaign ran between October 2021 and June 2022. The Australian project team was based in New South Wales and comprised a well-networked academic team including an old age psychiatrist, psychologist, nurse, occupational therapist and public health academic with experience in health campaigning.

#### Audience

The primary target audience for the campaign was clinicians involved in diagnosis of dementia (i.e. diagnosticians – geriatricians, old age psychiatrists, neurologists, clinical and neuro-psychologists) and professionals involved in providing post-diagnostic supports (i.e. dementia clinical nurse consultants, dementia advisors).

Key messages for professionals were: convey hope when telling someone they have dementia; recommend and initiate post-diagnostic support including use of the Forward with Dementia website; and provide a management plan, appropriate referrals and follow-up appointment, refer back to general practitioner (GP) for execution of management plan.

A secondary target audience was people with dementia, carers and family members who were active online. Key messages for people with dementia and carers were that**:** there are things you can do to live well with dementia; read information from the Forward with Dementia website; make a plan; and proactively seek supports and treatments to manage your dementia.

**Fig. 1:**
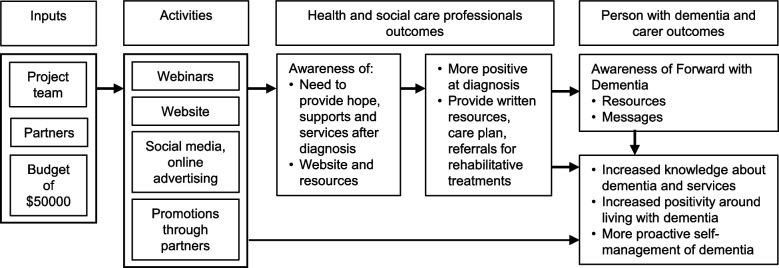
The Forward with Dementia campaign program logic

The campaign program logic is presented in Fig. 1.

#### Campaign activities

Key messages were promoted throughout the campaign via: webinars, website content, weekly website blog (e.g. stories from people living with dementia) and social media sharing (Twitter, Facebook, LinkedIn), monthly eNewsletters, media coverage of the launch, Google advertising, Facebook advertising, promotion through partner organisations’ (e.g. Dementia Australia, Royal Australian and New Zealand College of Psychiatrists) newsletters and events, and promotion via professional contacts.

People living well with dementia and carers participated in the online campaign as inspirational role models (including in a webinar panel discussion, providing news blogs and personal stories as well as strategies as to how they live well with dementia).

### Process evaluation

#### Design

A mixed methods process evaluation was undertaken. The Reach, Effectiveness, Adoption, Implementation, Maintenance (RE-AIM) framework [24] with the addition of appropriateness [25] was used to structure our evaluation. A description of the RE-AIM elements and data sources informing those elements are presented in Table 1.

**Table 1:** Process evaluation elements, description and data sources

Element	Description	Data sources^a^
Reach	Number of health and social care professionals, people with dementia and carers reached by the campaign, and how they were reached	C, D
Appropriateness	Perceived fit, relevance, compatibility, suitability and usefulness	A1, B, C
Effectiveness	Outcomes for health and social care professionals, people with dementia and carers	A1, A2, B, C, D
Adoption	Adoption or intended adoption of Forward with Dementia key messages by professionals and organisations and people with dementia and carers	A1, A2, B, C, D
Implementation	Fidelity to Forward with Dementia key messages by health and social care professionals	No relevant data
Maintenance	Forward with Dementia key messages and resources continue to be used and institutionalised	A2, C, D
Context	Factors relating to policy, regulations, and health care environments which affected the campaign	C, D

A1: Surveys of health and social care professionals—post-webinar

A2: Surveys of health and social care professionals—3–6 months post-engagement follow-up

B: Surveys of people with dementia and carers who had used the website

C: Interviews with health and social care professionals, people with dementia, carers, and key stakeholders

D: Log of campaign activities, engagements, and web analytics

^a^*Data sources*

#### Data collection

Data were collected during the eight months of campaigning (October 2021 – June 2022) and for three months following the campaign (July–September 2022).

Multiple types of data were collected for the evaluation:

A: Surveys of health and social care professionals
*A1: Post-webinar surveys*: Professionals who attended webinars were informed of the survey at the end of the webinar and subsequently invited to complete a short 5-min webinar evaluation survey by email. Survey respondents were presented with a screening question to clarify their role as a health and social care professional (or otherwise); health and social care professionals were led to the survey page with the Participant Information Statement as the preface, and submission of their survey was taken as an indication of their consent. Survey questions included how they learnt about the webinar, helpfulness of and information learnt from the webinar, if they had visited the website and if they intended to refer clients to the website.
*A2: 3–6 month post-engagement follow-up surveys*: Professionals were invited by email to complete a short 5-min survey between 3–6 months after attending a webinar, subscribing to the newsletter, or ordering printed campaign resources (e.g. leaflets, posters). Screening and consent process is the same as A1. The survey included a practice change tool [26, 27] adapted from use with nurses to apply to health and social care professionals. The tool is usually administered within 3–6 months of an educational intervention and measures change in one’s own thinking or practice and persuading others to change thinking or practice. Other survey questions included participation and usefulness of campaign, and use of Forward with Dementia website and resources.
B: Surveys of people living with dementia and carers who had used the website

People with dementia and carers who had visited the website were recruited through emails via the campaign mailing list, campaign newsletter, survey link on website, social media, StepUp for Dementia Research [28] and listing on the Dementia Australia website research page. Potential respondents who were interested were asked to click on the survey link included in the aforementioned channels, presented with the Participant Information Statement before the survey, and submission of their survey was taken as an indication of their consent.

The 20-min online survey included questions on helpfulness of the website and the campaign activities (e.g. webinars, podcast, social media posts, promotional materials, newsletters), and impact of the campaign on the participant’s understanding about services and supports for dementia and beliefs about dementia.

C:  Interviews with health and social care professionals, people with dementia, carers, and key stakeholders

A combination of purposeful and convenience sampling was undertaken. Survey participants from A and B above, after survey submission, were invited to participate in 45-min semi-structured, online interviews; those interested provided their contact details. Additional people with dementia and carers were recruited from StepUp for Dementia Research [28]. Key stakeholders (representatives of partner organisations, policy makers and dementia advocates who were engaged with the campaign) were recruited through personal invitation emails. Written informed consent was obtained from each interview participant.

The semi-structured interview questions related to experiences of campaign activities and perceived key messages of the Forward with Dementia campaign, as well as impact of this on the person’s knowledge, beliefs or behaviours, the organisation’s practices, or broader community. Data were transcribed verbatim by the research team (multiple members) or a professional transcriber.

D: Log of campaign activities, engagements, and web analytics

Campaign activities (e.g. webinars, advertisements, social media posts, newsletters, partner promotions) and engagements (e.g. attendees, reactions to social media posts, newsletter usage) were logged. Website usage data were obtained from Google Analytics.

#### Analysis

Descriptive analysis was undertaken of quantitative data from surveys using SPSS. Quantitative data are presented in the Results section as responses (numerator)/number responded to the individual question (denominator), with subscript ‘MD’ next to denominator to note missing data. Qualitative content analysis was undertaken of qualitative data from surveys' free-text responses and interviews. Relevant analysed data are triangulated and reported in Results using RE-AIM plus Appropriateness as a framework.

## Results

Overall, 81 health and social care professionals participated in the post-webinar survey (survey A1), 45 health and social care professionals participated in the post-engagement follow-up survey (survey A2), and 57 people with dementia or carers (15 and 42, respectively) participated in the website survey (survey B). Of those interviewed, eight were health and social care professionals, nine were people with dementia, six were carers and six were key stakeholders (a total of 29 interview participants).

Of the participants who actively provided information for the evaluation, 72% (149/207^MD^) were women. Health and social care professionals included representatives from all relevant professions and dementia support settings, and most states and territories. We had disproportionate participation from New South Wales at 46% (89/193^MD^). People with dementia and carers were relatively young (mean age of 68 ± 9 and 64 ± 11 years respectively). Characteristics of participants are presented in Table 2.

**Table 2:** Characteristic of evaluation participants

Characteristic	A: Surveys of health and social care professionals		B: Surveys of website users		C: Interviews				D: Google Analytics
	*A1: Post-webinar*	*A2: 3–6 months follow-up*	*People with dementia*	*Carers*	*Health and social care professionals*	*People with dementia*	*Carers*	*Key* *Stake-holders*	*Web users* (N = 3,009 users signed into their Google accounts)
*N*	81	45	15	42	8	9	6	6	11,920 visitors
*Mean* ± SD* (range)*	**Years in practice** (*n* = 77) 17.4 ± 10.4 (0–45)	**Years in practice** (*n* = 44) 16.2 ± 9.3 (1.5–35)	**Age** (*n* = 14) 67.9 ± 7.1 (54–79) **Years since diagnosis (** *n* = 13) 6.9 ± 3.8 (2–14)	**Age** 64.2 ± 10.6 (42–91) **Years since diagnosis** 3.8 ± 2.7 (0.3–10)	**Years in practice** 15.0 ± 8.1 (8–30)	**Age** 69.4 (54–84) **Years since diagnosis** 5.1 (1–10)	**Age** 67.3 (55–76) **Years since diagnosis** 3.4 (1–5)	NA	**Age** 45 (18–65 +)
*Women/Men/Missing data (% women)*	50/28/3 (64.1)	32/12/1 (72.7)	13/1/1 (92.9)	31/11/0 (73.8)	7/1 (87.5)	7/2 (77.8)	4/2 (66.7)	5/1 (83.3)	2,042/967 (67.9)
*State n (%)*									(*n* = 11,868)
* New South Wales*	38 (54.3)	18 (45.0)	4 (30.8)	20 (48.8)	5 (62.5)	5 (55.6)	6 (100.0)	3 (50.0)	5,152 (43.4)
* Victoria*	16 (22.9)	12 (30.0)	1 (7.7)	7 (17.1)	0	2 (22.2)	0	0	2,504 (21.1)
* Queensland*	5 (7.1)	4 (10.0)	2 (15.4)	8 (19.5)	2 (25.0)	0	0	0	1,780 (15.0)
* South Australia*	9 (12.9)	5 (12.5)	4 (30.8)	2 (4.9)	1 (12.5)	1 (11.1)	0	1 (16.7)	983 (8.3)
* Western Australia*	1 (1.4)	1 (2.5)	2 (15.4)	4 (9.8)	0	1 (11.1)	0	0	825 (7.0)
* Australian Capital Territory*	0	0	0	0	0	0	0	1 (16.7)	417 (3.5)
* Tasmania*	1 (1.4)	0	0	0	0	0	0	1 (16.7)	182 (1.5)
* Northern Territory*	0	0	0	0	0	0	0	0	25 (0.2)
* Missing data*	11	5	2	1					
*Setting n (%)* ^a^									
* Primary care*	3 (3.7)	1 (2.2)			4 (50.0)			0	
* Community*	21 (25.9)	16 (35.6)			3 (37.5)			0	
* Hospital*	36 (44.4)	18 (40.0)			2 (25.0)			1 (16.7)	
* Aged care home*	7 (8.6)	5 (11.1)			2 (25.0)			1 (16.7)	
* Private practice*	24 (29.6)	13 (28.9)			0			0	
* Memory clinic*	11 (13.6)	8 (17.8)			0			0	
* Other*	6 (7.4%)	9 (20.0%)	**Lived alone**: 3/14 (21.4)	**Lived with person with dementia**: 26/42 (61.9)	0	**Lived alone**: 2/9 (22.2)	**Lived with person with dementia**: 4/6 (66.7)	4 (66.7%)	**Device category** (*n* = 11,920) Desktop: 6,197 (52.0) Mobile: 4,383 (36.8) Tablet: 1,340 (11.2)

^a^Health and social care professionals were able to select more than one setting

### Reach

**Fig. 2:**
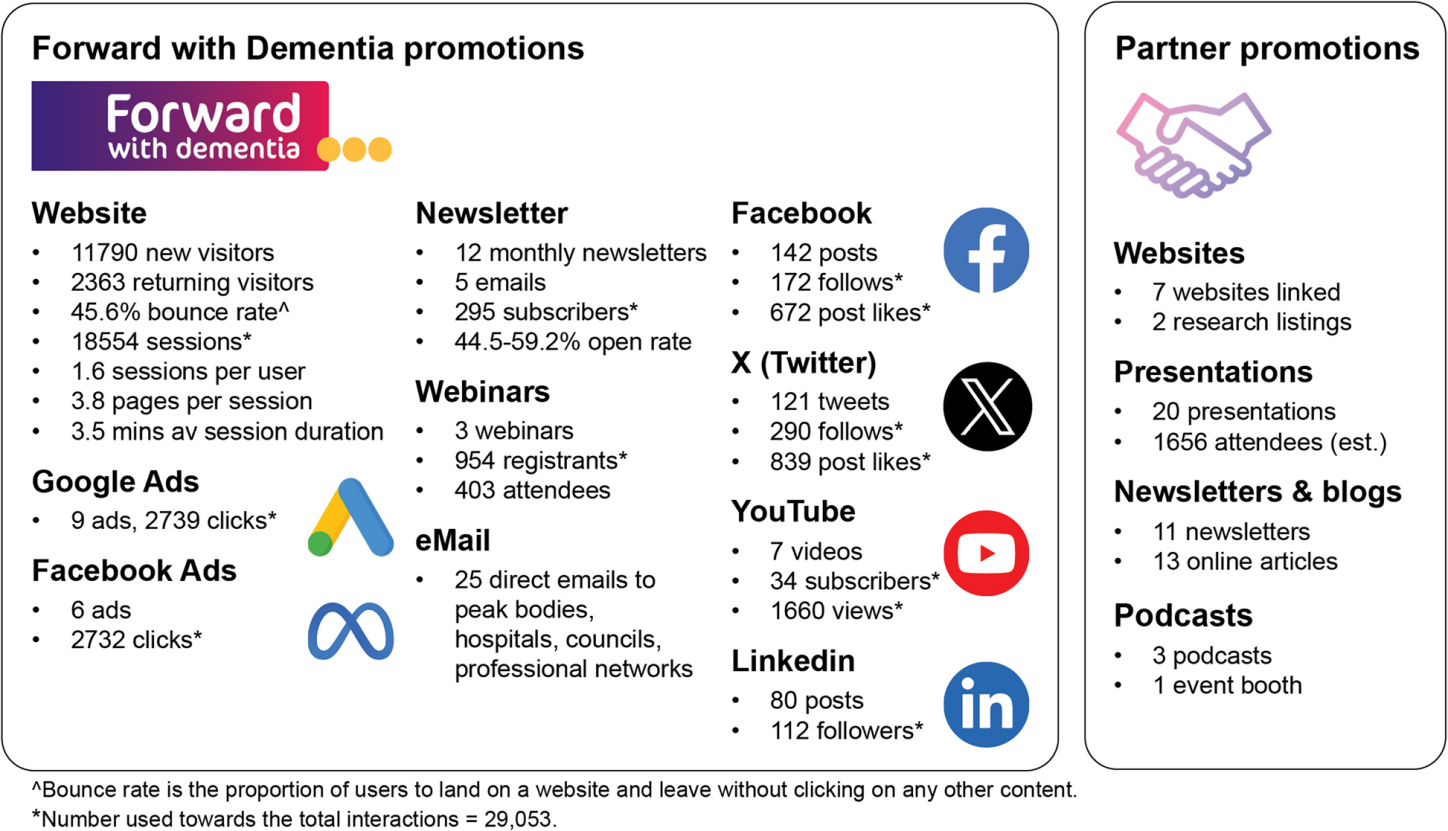
The Forward with Dementia campaign reach

Information regarding campaign reach between October 2021 and September 2022 is presented in Fig. 2. There were 29,053 interactions with campaign activities (e.g. browsing the website, liking a post, see Fig. 2). Some people had multiple interactions. The campaign was widely promoted through project partners, though we are not able to estimate reach through partner promotions. For most engagements, we had minimal information about the characteristics of the person engaging. In the 3 months post-campaign, there continued to be an average of 760 new visitors per month to the website.

### Appropriateness

More than three quarters (63/81; 78%) of Survey A1 respondents thought that information provided at the webinars was very or extremely helpful to their work in dementia care. A minority (3/81; 4%) said that they had already been providing the level of care covered in the webinars, which had therefore not added to their knowledge.

Health and social care professionals, people with dementia and carers described the website as having appropriate content: *“useful information”, “very enlightening”, “informative”, “thorough”, and “comprehensive”*. They also commented on the hopeful and friendly tone: *“relatable”, “I felt comfortable”, “positive and dynamic”,* and that it was easy to use*: “practical”, “self-explanatory”, “simple to navigate”, “user-friendly”, “well-structured”, “always there in case I forget things”*. One interviewee said they would have preferred someone to talk to instead of reading the website. A few health and social care professional interviewees and webinar attendees commented that the information was trustworthy based on the team’s academic background, expertise, and reputation.

“Your site has much more information, [compared to other websites] and it's much more sequential.” [person with dementia, Interviews C]

Of the 41/56^MD^ Survey B participants with dementia or carers who visited the Forward with Dementia website, 25/41 (61%) found the information very or extremely helpful, and 16/41 (39%) found the information slightly or moderately helpful. Of those Survey B participants who attended webinars or received newsletters, 18/25 (72%) and 13/25 (52%) respectively found them very or extremely helpful.

### Effectiveness

Health and social care practitioners described how Forward with Dementia increased their confidence in having challenging conversations around dementia and *“changing that narrative around it”.* Some also described positive impacts on their patients.

“I think he understood that it was not something that was going to get better, but he was looking for things to be positive about and it was the first person I think I really took that kind of hopeful approach with.” [health and social care professional, Interviews C].

“Forward with Dementia has utterly rescued me, but more importantly, my patients and carers who now come away from a visit much more hopeful and empowered” [health and social care professional, unsolicited feedback D].

People with dementia and carers in Survey B agreed or strongly agreed that Forward with Dementia improved their understanding of dementia (24/51^MD^, 47%) and what to expect in the future relating to dementia (24/50^MD^, 48%). However, they neither agreed nor disagreed that Forward with Dementia has given them information to help themselves live well (24/52^MD^, 46%), has helped them feel more confident to ask for professional help (27/52^MD^, 52%), has helped them feel more confident that they could handle changes that might occur in the future (25/52^MD^, 48%), or has helped them feel more confident that they can live with dementia in a positive way (25/52^MD^, 48%).

Specifically, Survey B carers agreed or strongly agreed that Forward with Dementia had given them information to help their person live well with dementia (23/38^MD^, 61%), and made it easier for them to find information about services/supports (18/39^MD^, 46%). However, Survey B people with dementia neither agreed nor disagreed that Forward with Dementia has helped them learn how to find information about services or supports (8/14^MD^, 57%), or helped them feel informed and prepared to live with dementia (7/13^MD^, 54%).

Some people with dementia described how the campaign led to decreases in self-stigma: *“it was like someone giving me a second chance”, “start to believe in myself more”, "my partner’s sense of agency has improved”.* People with dementia and carers also talked about improved wellbeing because of actions prompted by the campaign: “*ever since I've done it, I'm feeling 10 times, 100 times better than I originally was.”, “Exercise has engaged my partner in an activity (table tennis) that he really looks forward to”.* Carers found that the information helped them with their caring role by improving their understanding from the perspective of the person with dementia *“I’m getting into my mother’s head when I read that”*, giving them caring strategies *“I use it more for problem solving”*, and increasing their confidence as carers.

### Adoption

The majority of health and social care professionals said they were putting into action, or were planning to try actions aligned with the Forward with Dementia key messages. Ninety-six percent (77/80^MD^) of the professionals who completed the Survey A1 post-webinar planned to use learnings from the webinar in their work, 60/77^MD^ (78%) had visited the website and 75/79^MD^ (95%) intended to refer patients to the website. In qualitative comments, professionals described the webinar cultivated self-reflection on their professional practices: *“a really great catalyst”, “reinvigorating some interest”, “affirmation”, “opportunity to reflect”*.

Some professionals described they had changed the tone and content of information provided when communicating the diagnosis as a result of the campaign: “*giving people an element of hope and positivity while also trying to be realistic”, “instead of bringing doom to diagnosis, I can offer support and hope”.* They also described improvements in post-diagnostic management: “*I was able to offer a much more comprehensive plan than I had originally been trained to”.* They reported Forward with Dementia resources such as the Frequently Asked Questions for doctor and ‘circle of friends’ tool were useful: *“additional tools in my kit bag…”, “something tangible to sit down and talk with carers about”*.

Challenges to adoption were difficulties with making referrals for post-diagnostic services because of lack of services particularly in rural areas, lack of skilled professionals, difficult referral pathways and systemic silos, and therapeutic nihilism: “*You often hear people going, what’s the point*?”.

Only 41/56^MD^ (73%) of Survey B participants with dementia or carers had visited the website, even though it was an inclusion requirement, non-visitors were recruited through the StepUp for Dementia Research website. Of the participants who had visited, 17/41 (41%) has browsed through most of the material, and 7/41 (17%) had developed the suggested action plan. Reasons given for not developing the plan were that they already had one, they did not feel they needed this, it was too complicated, or they did not have the time.

### Maintenance

Of professionals surveyed 3–6 months post-engagement, 29/44^MD^ (66%) had changed some aspect of their own practice as a result of engaging with the campaign. 40/45 (89%) had educated a patient/carer, colleague or member of the public to make a change. 31/45 (69%) had persuaded a patient/carer, colleague or member of the public to make a change. A smaller proportion had changed a practice or routine in their unit or work area (17/44^MD^, 39%), changed a general non-clinical procedure/technique/intervention (17/43^MD^, 40%), changed a clinical policy/technique/intervention (12/43^MD^, 28%), or changed their beliefs about a particular approach/procedure (17/44^MD^, 39%). 14/45 (31%) of professionals reported that they had used the information or tools relating to Forward with Dementia often/very often in the last six months, 15/45 (33%) have used these some of the time in the last six months. Professionals reported recommending the website and sharing resources with their patients, “*printed it out and gave it to them*”, and disseminating to their colleagues: *“in my letter to the GP” “showed a colleague who missed the webinar*”. Twenty-six professionals requested printed resources be sent to them following the webinars. An average of 479 resources were downloaded per month after the active campaign ended (i.e. July–September 2022).

Some organisations are routinely using Forward with Dementia resources such as the ‘My Life Plan’ worksheet to support planning conversations, during the development of management plans, and when training GPs. Forward with Dementia has been promoted by multiple websites including Dementia Australia, Department of Health and Aged Care website for older persons, Department of Veterans Affairs, Australian Dementia Network, and Department of Health guidance for developing post-diagnostic pathways for Primary Healthcare Networks.

### Context

At the time of this campaign there were few professionals in Australia specifically funded to provide comprehensive post-diagnostic support. Memory clinics and public specialist clinicians were restricted in their ability to provide post-diagnostic care. Dementia Advisors were a role we had initially identified as providing post-diagnostic supports – however most of them had their roles revised and were no longer delivering such support as part of their work. Part of the campaign involved advocating for increased post-diagnostic provision by existing services.

The campaign was delivered during COVID restrictions – lockdowns lifted in most of Australia at the end of 2021. This was followed by rising rates of COVID infections, hospitalisations and death in early 2022 accompanied by continued precautions in health and aged care organisations. Because of this some health and social care practitioners had less capacity to engage with the campaign because of additional responsibilities.

## Discussion

The Forward with Dementia campaign was a relatively low budget, short, exclusively online program of activities with wide reach and appropriate content. The campaign resulted in adoption and maintenance of improved post-diagnostic support-related clinical practice in our primary audience of health and social care professionals. However, practice change was not universal, a minority of professionals reported already providing post-diagnostic support consistent with the campaign suggestions. Some professionals cited systemic barriers to helping their patients access treatments and services. This is consistent with research from the UK and Europe suggesting fragmented services without clear pathways, and with limited service capacity and capability [10, 29].

We anticipated being able to reach patients recently diagnosed and carers through participating clinicians, however inability to directly target, engage with, and recruit online and evaluate the impact of the campaign on our secondary audience of people recently diagnosed with dementia and their carers was a major weakness. The project team is based in NSW and were most able to engage with clinicians in NSW, further the majority of memory clinics are in urban areas, we would have less ability to reach people with dementia in other regions. Most people with dementia and carers surveyed and interviewed had known of their diagnosis well beyond our target period of 12 months after diagnosis, and some had not engaged with any campaign activities as they were recruited through StepUp for Dementia Research, a research recruitment initiative [28]. A known barrier to recruitment of people with dementia and carers is their needing time to adjust to their diagnosis before being ready to volunteer for research [30]. Hence, we do not know if the campaign was efficacious in terms of increasing the knowledge and behaviours of recently diagnosed people with dementia and carers.

A second limitation of our evaluation was the relatively short campaign of eight months and evaluation follow-up of professionals of 3–6 months. Participants and organisations might not have had enough time to embed campaign recommendations and resources into practice. Another limitation was participant bias, as it is plausible professionals who were more willing to change practices were also more willing to participate in the evaluation. Strengths of this evaluation are the use of multiple data collection methods, samples and data triangulation, which increases our confidence in the findings.

Messages and resources were ensured to be appropriate and persuasive for the target audiences, these were developed through a co-design process. The co-design process also resulted in the use of a hopeful and positive tone throughout the campaign, this may have contributed to reducing dementia stigma [31]. Inclusion of people living well with dementia as part of the campaign has been shown to be an effective strategy in dementia friendly campaigns [22] and reduce stigma [32]. The respected reputation and network of the campaign team enabled wide promotion through project partners. This campaign differed from one-off training webinars as ongoing promotion through multiple channels supported frequent and continuous exposure to the messages over time which is important for behaviour change [31].

Future campaigns could consider physical promotion (e.g. posters and flyers in public places) and other outdoor advertising, as well as in-person events to increase adoption, maintenance and effectiveness. Future evaluations might utilise ripple effect mapping to capture indirect impacts achieved through participants acting on and sharing campaign messages [33].

## Conclusions

In conclusion, this campaign to improve the diagnostic conversation for dementia positively influenced professional practice though the evaluation was unable to demonstrate flow-through benefits for people living with dementia and carers. A longer campaign and targeted engagement and recruitment of recipients of post-diagnostic supports would improve both the campaign and evaluation.

## Data Availability

The datasets generated and/or analysed during the current study are not publicly available due to ethical restrictions and participant confidentiality, and are available from the corresponding author upon reasonable request.

## Abbreviations

GP: General practitioner
RE-AIM: Reach, effectiveness, adoption, implementation, maintenance
UK: United Kingdom

## References

[1] Gauthier S, Webster C, Servaes S, Morais JA, Rosa-Neto P (2022). World Alzheimer Report 2022 Life after diagnosis: Navigating treatment, care and support.

[2] Bamford C, Wheatley A, Brunskill G, Booi L, Allan L, Banerjee S, et al. Key components of post-diagnostic support for people with dementia and their carers: A qualitative study. PLoS ONE. 2021;16(12 December):e0260506. 10.1371/journal.pone.0260506 PMC8687564 34928972

[3] Low LF, Gresham M, Phillipson L (2023). Further development needed: models of post-diagnostic support for people with dementia. Curr Opin Psychiatry.

[4] Low LF (2019). Why Australia urgently needs post-diagnostic support and treatment for dementia. Aust J Dementia Care.

[5] Naismith SL, Michaelian JC, Low LF, Arsenova V, Mehrani I, Fyfe K (2022). Characterising Australian memory clinics: current practice and service needs informing national service guidelines. BMC Geriatr.

[6] Arblaster K, Brennan S (2022). Left to cope alone: the unmet support needs after a dementia diagnosis.

[7] Stephan A, Bieber A, Hopper L, Joyce R, Irving K, Zanetti O, et al. Barriers and facilitators to the access to and use of formal dementia care: findings of a focus group study with people with dementia, informal carers and health and social care professionals in eight European countries. BMC Geriatr. 2018;18(1). 10.1186/s12877-018-0816-1 PMC5987478 29866102

[8] Mansfield E, Bryant J, Nair BR, Zucca A, Pulle RC, Sanson-Fisher R (2022). Optimising diagnosis and post-diagnostic support for people living with dementia: geriatricians’ views. BMC Geriatr.

[9] Giebel C, Verbeek H, Robertson S, Beaulen A, Zwakhalen S, Allen D (2021). “Nobody Seems to Know Where to Even Turn To”: Barriers in Accessing and Utilising Dementia Care Services in England and The Netherlands. Int J Environ Res Public Health.

[10] Wheatley A, Bamford C, Brunskill G, Booi L, Dening KH, Robinson L (2021). Implementing post-diagnostic support for people living with dementia in England: A qualitative study of barriers and strategies used to address these in practice. Age Ageing.

[11] Frost R, Walters K, Wilcock J, Robinson L, Harrison Dening K, Knapp M (2021). Mapping post-diagnostic dementia care in England: an e-survey. J Integr Care.

[12] Low LF, Laver K, Lawler K, Swaffer K, Bahar-Fuchs A, Bennett S (2021). We need a model of health and aged care services that adequately supports Australians with dementia. Med J Aust.

[13] Cations M, May N, Crotty M, Low LF, Clemson L, Whitehead C (2020). Health professional perspectives on rehabilitation for people with dementia. Gerontologist.

[14] Milne A (2010). The 'D' word: Reflections on the relationship between stigma, discrimination and dementia. J Ment Health (Abingdon, England).

[15] World Health Organisation (2018). Ensuring a human rights-based approach for people living with dementia.

[16] Van Den Dungen P, Van Kuijk L, Van Marwijk H, Van Der Wouden J, Moll Van Charante E, Van Der Horst H, et al. Preferences regarding disclosure of a diagnosis of dementia: a systematic review. Int Psychogeriatr. 2014;26(10):1603–18. 10.1017/S1041610214000969 24933479

[17] Poyser CA, Tickle A (2019). Exploring the experience of the disclosure of a dementia diagnosis from a clinician, patient and carer perspective: a systematic review and Meta-ethnographic synthesis. Aging Ment Health.

[18] Van Asbroeck S, van Boxtel MPJ, Steyaert J, Köhler S, Heger I, de Vugt M (2021). Increasing knowledge on dementia risk reduction in the general population: Results of a public awareness campaign. Prev Med.

[19] Talbot LA, Thomas M, Bauman A, Manera KE, Smith BJ (2021). Impacts of the national your brain matters dementia risk reduction campaign in Australia Over 2 Years. J Alzheimer's Dis.

[20] Heger I, Köhler S, Van Boxtel M, De Vugt M, Hajema K, Verhey F (2020). Raising awareness for dementia risk reduction through a public health campaign: a pre-post study. BMJ Open.

[21] Hung L, Hudson A, Gregorio M, Jackson L, Mann J, Horne N (2021). Creating dementia-friendly communities for social inclusion: A scoping review. Gerontol Geriatr Med.

[22] Phillipson L, Hall D, Cridland E, Fleming R, Brennan-Horley C, Guggisberg N (2019). Involvement of people with dementia in raising awareness and changing attitudes in a dementia friendly community pilot project. Dementia.

[23] Zheng J, Gresham M, Phillipson L, Hall D, Jeon YH, Brodaty H, et al. Exploring the usability, user experience and usefulness of a supportive website for people with dementia and carers. Disabil Rehabil Assist Technol. 2023:1–13. 10.1080/17483107.2023.2180546. Epub ahead of print. 10.1080/17483107.2023.2180546 37086036

[24] Holtrop JS, Estabrooks PA, Gaglio B, Harden SM, Kessler RS, King DK (2021). Understanding and applying the RE-AIM framework: Clarifications and resources. J Clin Transl Sci.

[25] Proctor E, Silmere H, Raghavan R, Hovmand P, Aarons G, Bunger A (2011). Outcomes for implementation research: conceptual distinctions, measurement challenges, and research agenda. Adm Policy Ment Health.

[26] Estabrooks CA, Floyd JA, Scott-Findlay S, O'Leary KA, Gushta M (2003). Individual determinants of research utilization: a systematic review. J Adv Nurs.

[27] Estabrooks CA (1999). The conceptual structure of research utilization. Res Nurs Health.

[28] Jeon Y-H, Shin M, Smith A, Beattie E, Brodaty H, Frost D (2021). Early Implementation and evaluation of stepup for dementia research: an australia-wide dementia research participation and public engagement platform. Int J Environ Res Public Health.

[29] Martin A, O’Connor S, Jackson C (2020). A scoping review of gaps and priorities in dementia care in Europe. Dementia.

[30] Field B, Mountain G, Burgess J, Di Bona L, Kelleher D, Mundy J (2019). Recruiting hard to reach populations to studies: breaking the silence: an example from a study that recruited people with dementia. BMJ Open.

[31] Wakefield MA, Loken B, Hornik RC (2010). Use of mass media campaigns to change health behaviour. Lancet.

[32] Kim S, Richardson A, Werner P, Anstey KJ (2021). Dementia stigma reduction (DESeRvE) through education and virtual contact in the general public: A multi-arm factorial randomised controlled trial. Dementia (London).

[33] Workman LM, Browder JS (2020). The Use of Ripple Effect Mapping to Understand Successes of the SC Pregnancy Assistance Fund: A Participatory Evaluation Approach. Matern Child Health J.

